# Vernier Effect-Enhanced Temperature Sensing Based on On-Chip Spiral Resonant Cavities

**DOI:** 10.3390/s25030685

**Published:** 2025-01-23

**Authors:** Changhao Liu, Ziwen Pan, Yi Yang, Xi Yang, Jun Tang

**Affiliations:** 1 Concord College, Acton Burnell Hall, Acton Burnell, Shrewsbury, Shropshire SY5 7PF, UK; changhaoliu11@foxmail.com; 2 School of Instrument and Electronics, North University of China, Taiyuan 030051, China; panziw@foxmail.com (Z.P.); yangyinuc@163.com (Y.Y.); 3 School of Politics and Public Administration, University of Electronic Science and Technology of China, Chengdu 611731, China; 4 School of Physics, Peking University, Beijing 100871, China; 5 School of Semiconductors and Physics, North University of China, Taiyuan 030051, China

**Keywords:** whispering gallery mode (WGM) resonant cavity, Vernier effect, temperature sensing

## Abstract

The optical Vernier effect has been widely studied due to its remarkable effect in improving the sensitivity and resolution of optical sensors. This effect relies on the overlapping envelope of two signals with slightly detuned frequencies. In the application of on-chip optical waveguide resonant cavities with whispering gallery modes, due to the on-chip space limitations, the length of the resonant cavity is restricted, resulting in an increased free spectral range. In the case of a small Vernier effect detuning, the required large Vernier envelope period often exceeds the available wavelength range of the detection system. To address this issue, we propose a novel on-chip waveguide structure to optimize the detection range of the cascaded Vernier effect. The proposed spiral resonant cavity extends the cavity length to 7.50 m within a limited area. The free spectral width (27.46 MHz) is comparable in size to the resonant linewidth (9.41 MHz), shrinking the envelope free spectral width to 371.29 MHz, which greatly facilitates the reading of the Vernier effect. Finally, by connecting two resonant cavities with similar cavity lengths in series and utilizing the Vernier effect, temperature sensing was verified. The results show that compared with a single resonant cavity, the sensitivity was improved by a factor of 14.19. This achievement provides a new direction for the development of wide-range and high-sensitivity Vernier sensing technologies.

## 1. Introduction

The optical Vernier effect is a new method for enhancing the sensitivity and resolution of optical sensors. This effect relies on the overlapping envelopes of two signals with slightly detuned interfering frequencies, which can effectively magnify the wavelength shift in the interference spectrum, thus significantly improving the measurement sensitivity [1,2,3]. With the rapid development of optical sensor technology, the optical Vernier effect has been verified on various optical platforms, such as Fabry–Perot interferometers [4,5], Mach–Zehnder interferometers [6,7,8], Sagnac interferometers [9,10], Michelson interferometers [11,12], etc. It can convert small physical quantity changes into easily detectable optical signal changes, enabling precise measurement of these parameters. For example, in temperature sensing, high-precision monitoring of environmental temperature can be achieved by monitoring the wavelength shift in the interference spectrum [13,14]. In refractive index sensing, the optical Vernier effect can be used to detect minor changes in the refractive index of media [15,16,17], which is of great significance for fields such as biomedicine [18,19] and chemical analysis [20,21].

At present, the optical Vernier effect is mainly realized on the fiber sensing platform. Yunhao Chen pointed out in his review of fiber sensors based on this effect that by accurately matching the free spectral widths of two fiber interferometers, the sensitivity of a single fiber sensor can be significantly improved [22]. Specifically, the closer the free spectral widths of the two fiber interferometers are, the larger the magnification factor is. However, there is a certain trade-off between the magnification factor and the Vernier envelope: a larger magnification factor is often accompanied by an increase in the Vernier effect envelope, which makes precise detection more difficult. In view of the actual needs of small space or system integration, researchers have explored the application of the optical Vernier effect in on-chip systems through micro-nano processing technology, significantly reducing the volume and weight of the equipment. At the same time, by optimizing the waveguide structure, the sensitivity of the system has been further improved, and the sensing range has been broadened. Tong Chen proposed a high-sensitivity refractive index sensor [23]. Compared with a single micro-ring resonator, this sensor shows higher refractive index sensitivity based on the Vernier effect. By optimizing the resonator parameters, the refractive index sensing range of the sensor has been successfully expanded.

Nonetheless, most current cascaded sensing platforms based on the on-chip optical Vernier effect still face challenges. Due to the limitations of the calculation instruments and processes, envelope reading has become particularly difficult. Wassana Naku pointed out in his research that the sensing detection based on the Vernier effect relies on two interferometers with slightly detuned FSR to achieve ultra-high-sensitivity measurement. However, this measurement method faces the challenge that the increase in sensitivity leads to a large-and-difficult-to-identify Vernier envelope. In addition, due to the limited wavelength observation bandwidth of the instrument, it is difficult to accurately quantify the envelope signal [24].

In this paper, a temperature sensor based on an on-chip spiral resonant cavity is proposed. Based on micro-nano processing technology, through the design of multiple turn structures of the resonant cavity, the cavity length is increased to 7.50 m within an effective area. The free spectral width (FSR) of the resonant cavity is precisely regulated to a level comparable to its resonant linewidth, offering a way for the effective reading of the envelope signal. Finally, the temperature sensing is verified by using the Vernier effect of two resonant cavities with similar cavity lengths in series. The temperature sensitivity of the final envelope is 23.84 GHz/°C, which is 14.19 times higher than that of a single resonant cavity. The research content shows enormous potential in optical sensing performance and provides new ideas for the design of high-precision and high-sensitivity optical sensors in the future.

## 2. Principle

**Figure 1:**
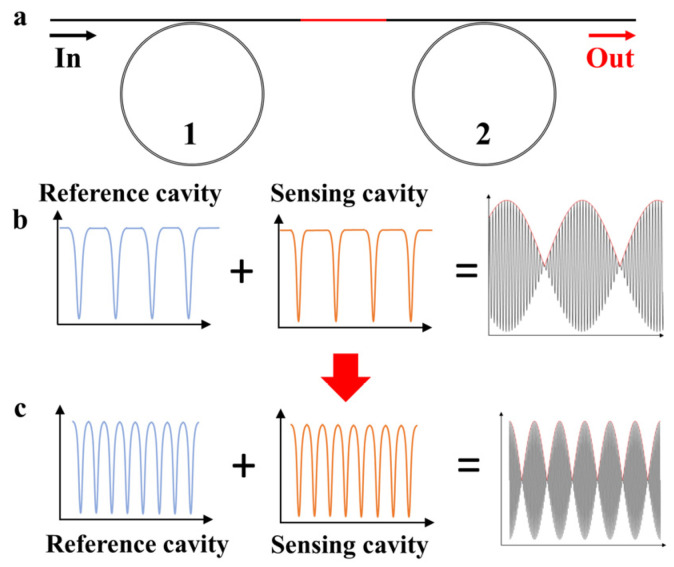
Schematic of the sensing principle of the Vernier effect. (**a**) Schematic of the Vernier effect system with two on-chip whispering gallery mode resonant cavities cascaded. (**b**) Schematic of the FSR and envelope curve of the on-chip single-loop cascaded resonant cavity. (**c**) Schematic of the cascaded spiral waveguide for enhancing the reading ability of the system.

The Vernier effect was initially applied to improve the resolution of length measurements, such as in Vernier calipers. It utilizes the slight scale difference between the main scale and the Vernier scale and magnifies and reads the small changes in measured values through the aligned scale markings of the Vernier scale and the main scale, thus enhancing the accuracy of length measurement. In the optical field, the Vernier effect is often used to improve the sensitivity of sensors. When two devices with slightly different free spectral ranges are cascaded, the optical Vernier effect can be formed (Figure 1a). This effect usually depends on the combination of two interferometers. Analogous to the Vernier caliper, their interference signals are regarded as the fixed scale and the Vernier scale. When the two interference signals have slightly shifted interference frequencies, the two signals will superimpose to produce envelope modulation. When the spectrum of one of the sensors has a small wavelength shift due to the perturbation of the measured quantity, the superimposed envelope will have a large-scale wavelength shift, thereby amplifying and reading the small wavelength shift.

In the Vernier effect, the envelope range and the amplification factor M are two very important parameters, which have a direct impact on the realization and performance of the Vernier effect. When light waves are repeatedly reflected in the resonant cavity to form resonance, a series of discrete longitudinal modes will be formed. The interval between these longitudinal modes is the free spectral range (FSR). In the whispering gallery mode resonant cavity, the FSR of a single resonant cavity is directly determined by the cavity length:

(1) FSR=c/nL

where *c* is the speed of light in vacuum, *n* is the refractive index of the waveguide medium, and *L* is the length of the resonant cavity. When the change in external physical quantity causes the effective refractive index or the cavity length and other parameters of the sensing cavity to change, and then leads to a change in wavelength, the interference patterns generated by the two cascaded resonant cavities will be superimposed on each other to form an interference envelope. The frequency or wavelength interval corresponding to the periodically repeated part of this envelope is the free spectral range of the envelope (Figure 1b):

(2) $${\mathrm{FSR}}_{\mathrm{envelope}}=\left|\frac{{\mathrm{FSR}}_{2}{\mathrm{FSR}}_{1}}{{\mathrm{FSR}}_{2}-{\mathrm{FSR}}_{1}}\right|$$

where FSR_1_ is the FSR of the sensing cavity and FSR_2_ is the FSR of the reference cavity. FSR_envelope_ determines the laser scanning range required in the reading process. At the same time, the ratio between the FSR_envelope_ of the Vernier envelope and the FSR_1_ of the sensing interferometer is defined as the amplification factor *M*. The size of M directly determines the measurement sensitivity of the Vernier effect.

(3) $$M=\frac{{\mathrm{FSR}}_{\mathrm{envelope}}}{{\mathrm{FSR}}_{1}}=\frac{{\mathrm{FSR}}_{2}}{{\mathrm{FSR}}_{2}-{\mathrm{FSR}}_{1}}$$

It can be seen from Equation (3) that when FSR_1_ and FSR_2_ are close, the system can obtain a large amplification factor, but at the same time, according to Equation (2), the FSR_envelope_ will increase greatly. Especially in the application of high-*Q* whispering gallery mode resonant cavities, due to the high *Q* value of the resonant cavity, a tunable narrow-linewidth laser is often used for resonant spectrum scanning. Currently, the fine laser scanning range is often limited to the GHz to tens of GHz range by devices [25]. However, the shorter cavity length under the limited space usually leads to the FSR of the resonant cavity being above the GHz order of magnitude. This relatively large FSR_envelope_ under the limited reading ability of the instrument leads to a great limitation of the response range and reading accuracy, and it is difficult to achieve a wide range of reading and measurement of the sensing signal. Therefore, balancing the cavity length difference and the envelope period is the key to improving the system accuracy and usability, and designing a reasonable cavity length is crucial for improving the system performance.

To increase the cavity length and improve performance in a limited area, we propose an on-chip spiral resonant cavity structure. This design effectively increases the cavity length through the on-chip spiral structure and avoids the scattering crosstalk caused by the crossed waveguides. By strictly controlling the cavity length design and the precision processing technology, the FSR is reduced to be close to the resonant spectral linewidth, thereby improving the readability of the envelope. As shown in Figure 1c, this scheme not only achieves a large reading range but also has a significant amplification factor. This design method provides a reliable solution for high-sensitivity sensing and high-precision signal reading.

## 3. Experiment

Traditional on-chip waveguide resonant cavities are usually designed as single-loop structures such as ring or racetrack shapes due to process limitations. Such structures are convenient for coupling and have relatively low bending losses, enabling the design of resonant cavities with high *Q* values. Equation (4) presents the approximate relationship between the input and output light intensities of the optical field in a ring resonant cavity [26].

(4) $$T=\frac{I_{out}}{I_{in}}={\left|\frac{E_{out}}{E_{in}}\right|}^{2}=\frac{t^{2}+a^{2}-2ta\cos\phi }{1+t^{2}a^{2}-2ta\cos\phi }$$

where *I*_out_/_in_ and *E*_out_/_in_ represent the input and output light intensities and electric field intensities of the resonant cavity, respectively, *t* represents the transmission coefficient, and a represents the round-trip coefficient. It can be seen from Equation (4) that the transmission coefficient t and the round-trip coefficient *a* are the key parameters affecting the resonant spectrum of the system. The transmission coefficient *t* can be adjusted by changing the coupling spacing. When the resonant cavity is in different coupling states, the quality factor and the resonant depth will change. The round-trip coefficient a is related to factors such as the shape, size, material properties, and fabrication process of the waveguide. The roughness, curvature, and existing defects on the waveguide surface may all have an impact on the propagation of light and thus affect the round-trip coefficient.

To obtain a better Vernier effect, cascaded resonant cavities need to be designed with resonant spectra having high *Q* values and large resonant depths while maintaining a small FSR, which poses high requirements for the design of resonant cavities.

A higher quality factor determines that the cavity can store and amplify optical signals more effectively, which helps to enhance the intensity of the output signal. Meanwhile, a narrower linewidth can improve the system resolution. The *Q* value can be expressed as follows:

(5) $$Q=\frac{n\pi L}{\lambda \arccos\frac{2ta}{1+t^{2}a^{2}}}$$

The resonant depth is a quantitative index that describes the attenuation degree of the optical signal in the resonant cavity and reflects the depth of the resonant trough compared to the resonant peak. The resonant depth can be expressed as follows:

(6) $$h=1-{\left(\frac{t-a}{1-ta}\right)}^{2}$$

Affected by processing technology, when designing resonant cavities, unit losses and bending losses usually need to be considered. Through the actual measurement of process parameters in the early stage [27], for the silica waveguide we selected, the refractive indices of the core layer and the cladding layer are *n*_1_ = 1.456 and *n*_2_ = 1.445, respectively. The unit transmission loss is approximately 0.005~0.008 dB/cm, the bending loss is compensated by 0.1 dB per 90° at a radius of 5 mm, and the size of the waveguide core is approximately 6 × 6 μm^2^ to ensure single-mode transmission of the laser with a wavelength of 1550 nm.

**Figure 2:**
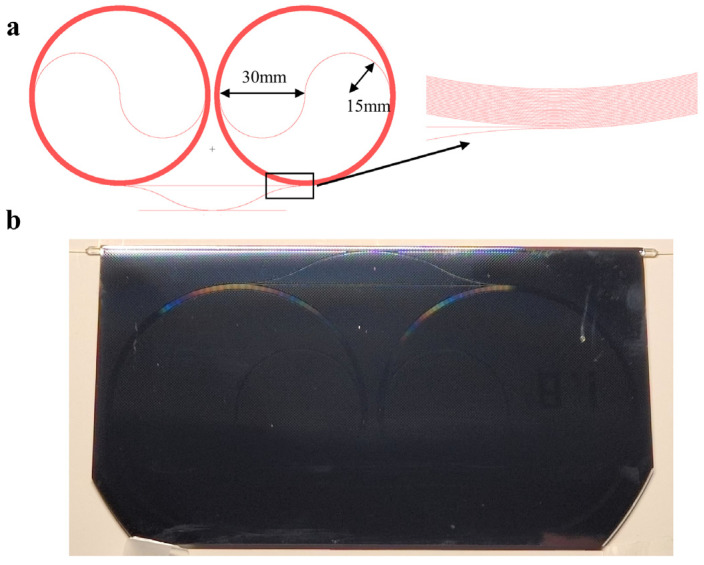
A schematic design and actual image of an on-chip spiral waveguide resonator. (**a**) shows the design layout of the resonator. (**b**) shows the actual image of the prepared resonator.

To prepare a larger cavity length within a limited area to reduce the FSR, we first designed the spiral waveguide structure shown in Figure 2a. This structure is formed by connecting two completely symmetrical spiral structures, avoiding waveguide crossings. The minimum radius at the inner ring connection is approximately 15 mm, the outer ring radius is approximately 30 mm, and the overall length is approximately 7.5 m. This cavity length ensures that the FSR is slightly larger than the linewidth determined by the theoretical *Q* value, preventing the superposition of resonant spectra when the FSR is lower than the linewidth of the resonant cavity. Through numerical calculation, the designed coupling spacing is approximately 1.8 μm to ensure the maximization of the resonant depth.

**Figure 3:**
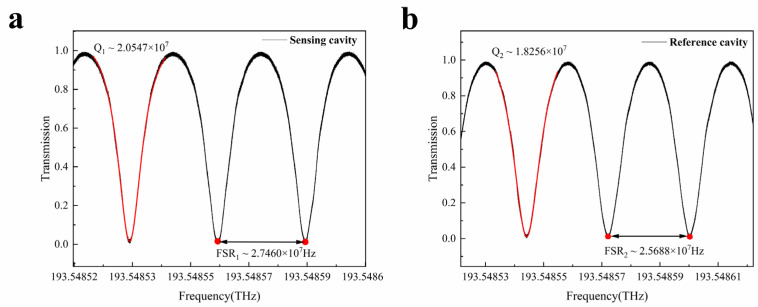
Measurement results of resonant cavity parameters. (**a**) Measurement results of resonant spectrum line parameters of the sensing cavity. (**b**) Measurement results of resonant spectrum line parameters of the reference cavity.

The image of the designed waveguide cavity is shown in Figure 2b. A 1550 nm tunable external cavity diode laser (ECDL) was used to evaluate the resonant spectrum parameters of the resonant cavity. By applying a triangular wave modulation signal to the laser, the wavelength scanning of the resonant cavity was achieved to excite its matched resonant modes. The observed resonant spectrum is shown in Figure 3a, with a *Q* value of approximately 2.05 × 10^7^ and an FSR of approximately 2.746 × 10^7^ Hz.

To excite the optical cascaded Vernier effect, we adopted this cavity as the sensing cavity and designed a reference cavity with the same structural design as the cascading object. The designed reference cavity has a designed cavity length of approximately 8.05 m by slightly adjusting the number of turns and the radius. The test results of its resonant spectrum are shown in Figure 3b, with a *Q* value of approximately 1.83 × 10^7^ and an FSR of approximately 2.569 × 10^7^ Hz.

The spectra of the two designed resonant cavities have obvious single-mode periodicity, and the resonant depth is close to 100%. The FSR values are slightly larger than the full width at half maximum of the spectra, and they have a small difference, which is suitable for cascaded Vernier effect sensing.

**Figure 4:**
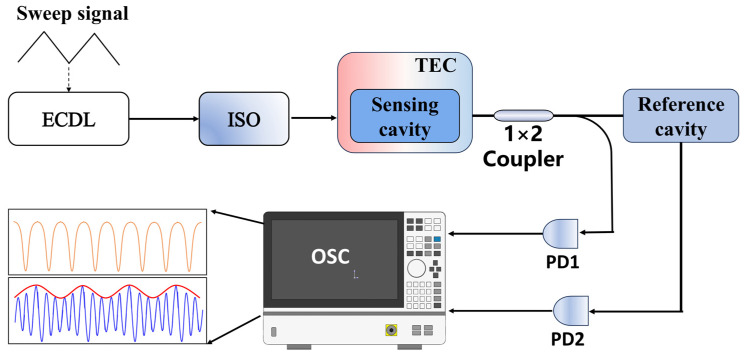
Schematic of the temperature testing system for the cascaded Vernier effect.

Figure 4 shows the schematic diagram of the temperature testing system. A tunable external cavity diode laser with a wavelength of 1550 nm was used as the incident light source, and the laser output power was set at 10 mW. After passing through an optical isolator (ISO), the laser entered the sensing resonant cavity. The sensing resonant cavity was encapsulated in a temperature control device using thermal insulation materials, and a thermoelectric cooler (TEC) was employed to control the temperature of the sensing cavity. To record and monitor the changing trends of the sensing cavity and the Vernier envelope, a 1 × 2 coupler with a coupling ratio of 50:50 was used in the optical path. One output light passed through photodetector PD_1_, and the collected optical signals were converted into electrical signals and sent to an oscilloscope for single-cavity temperature sensing measurement. The other output light was cascaded with the reference waveguide cavity and then passed through photodetector PD_2_, and the signals were sent to the oscilloscope for Vernier envelope measurement.

**Figure 5:**
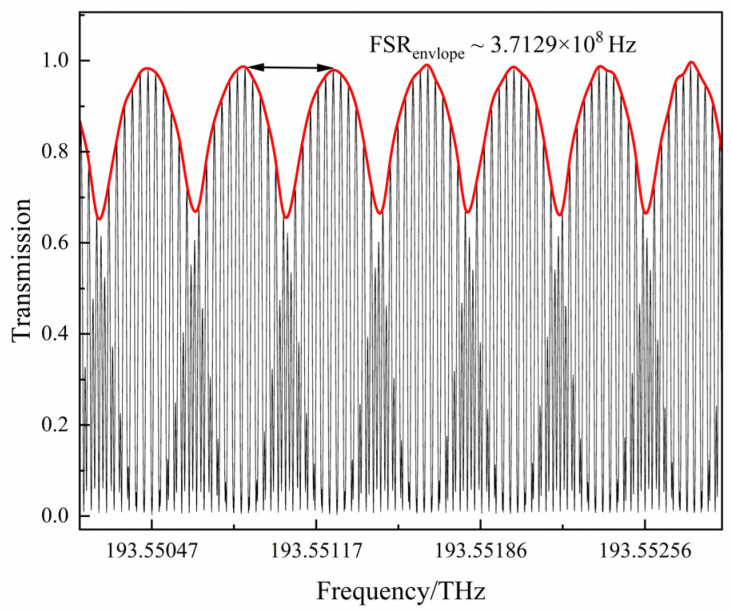
Test results of the envelope curve of the cascaded resonant cavities.

Firstly, the Vernier envelope results output by PD_2_ were tested under room temperature conditions. Figure 5 shows the fitting results of the superimposed spectra and their envelopes. The black curve is the superposition of spectra, and the red curve shows the fitting result of the envelope. The measured FSR_envelope_ is 3.7129 × 10^8^ Hz. According to Equation (2), the theoretically calculated FSR_envelope_ is approximately 3.9791 × 10^8^ Hz. The two values have a relatively high degree of agreement, which verifies the reliability of the theoretical model. Under these parameter conditions, the theoretical amplification factor *M* is approximately 14.49. Compared with the racetrack-shaped single-loop resonant cavity (with a cavity length of approximately 32 cm) under the same shape, under the same amplification factor, its envelope FSR will reach 10 GHz, which is approximately 27 times larger than the envelope FSR of our proposed method.

**Figure 6:**
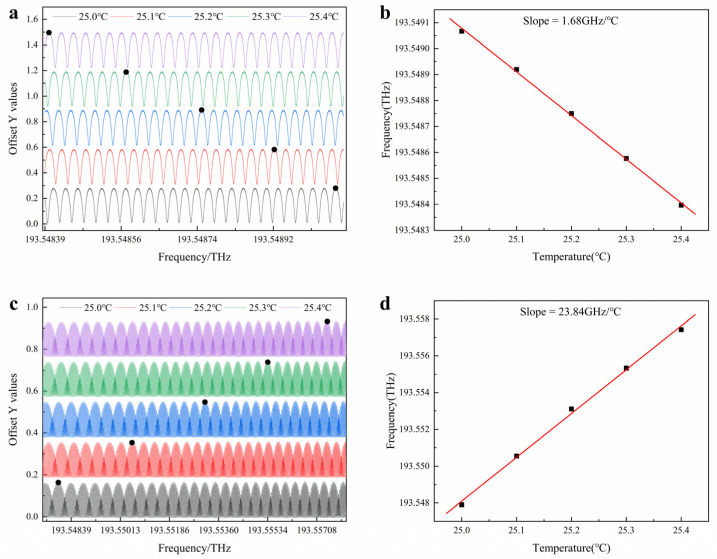
Temperature test results of the single resonant cavity and the cascaded Vernier system. (**a**) Test results of the resonant spectrum line of the single sensing cavity varying with temperature. Note: *Y*-axis units (offset Y values) are dimensionless and show the offset of each data set relative to the *Y*-axis zero point. (**b**) Fitting results of the temperature sensitivity of the single sensing cavity. (**c**) Test results of the cascaded Vernier envelope curve varying with temperature. (**d**) Fitting results of the temperature sensitivity of the cascaded Vernier envelope.

By using the temperature control device to change the ambience temperature of the cavity, the drift situation of the resonant spectrum line of the sensing cavity output by PD1 was recorded in real time, and the temperature response characteristics of the sensing cavity were tested, as shown in Figure 6a. The test step temperature difference was set as a fixed value (0.1 °C) to ensure sufficient resolution and data point density. The test results show that the resonant spectrum line gradually drifts under the influence of temperature and has good linearity. By measuring the position of the resonant peak at preset temperature points and drawing a linear fitting curve with the wavelength drift amount (Δ*f*) against the temperature change amount (Δ*T*), the experimentally measured temperature sensitivity of a single sensing cavity is approximately 1.68 GHz/°C, as shown in Figure 6b.

To verify the response of the cascaded Vernier effect to temperature sensitivity, the cascaded output of the sensing cavity and the reference cavity was measured. In the cascade system, the sensing cavity was placed in the same adjustable temperature-controlled environment, and its temperature response was tested under the same test conditions (temperature range and step value). The test results are shown in Figure 6c. The envelope curve shows a drift in the opposite wavelength with the change in temperature, which is caused by the comparison of the FSR sizes of the two cascaded cavities. According to Equation (3), when the relationship between the FSR of the reference cavity and that of the sensing cavity is different, the drift direction of the envelope is also reversed. By accurately measuring the variation in the position of the envelope peak at different temperatures, drawing the relationship curve between the envelope drift amount and the temperature change, and performing linear fitting, the temperature sensitivity of the envelope is approximately 23.84 GHz/°C. Compared with the sensitivity of a single sensing cavity, it has been enhanced by a factor of 14.19. This indicates that the cascaded structure enhances the temperature sensitivity, while ensuring the readability of the small envelope and significantly increasing the resolution ability of the system.

## 4. Conclusions

In this paper, an on-chip waveguide resonant cavity with a spiral structure design is proposed. Through strict design and process requirements, the length of the resonant cavity is increased within an effective area, reducing the FSR to 27.46 MHz while keeping a relatively high optical *Q* value with a full width at half maximum reaching 9.41 MHz. Through the temperature comparison test of the cascaded Vernier envelope, the sensitivity has been significantly improved, reaching 23.84 GHz/°C, which is 14.19 times higher than that of a single resonant cavity. These results verify the potential of the cascaded optical Vernier effect of on-chip waveguide resonant cavities in high-sensitivity and wide-dynamic-range temperature sensing, providing reliable theoretical and experimental bases for practical applications. The future combination of Vernier effect with nonlinear effects may achieve more efficient signal enhancement and tuning within the frequency domain, promising more opportunities for integrated photonics and frequency conversion applications [28].

## Data Availability

Data are contained within the article.
